# USP14 exhibits high expression levels in hepatocellular carcinoma and plays a crucial role in promoting the growth of liver cancer cells through the HK2/AKT/P62 axis

**DOI:** 10.1186/s12885-024-12009-y

**Published:** 2024-02-21

**Authors:** Nannan Zhang, Hui Zhang, Xiaobing Yang, Qiang Xue, Quhui Wang, Renan Chang, Lirong Zhu, Zhong Chen, Xiancheng Liu

**Affiliations:** 1 Medical College of Nantong University, Nantong, Jiangsu 226000 China; 2 Department of General Surgery, Affiliated Hospital of Nantong University, Nantong, Jiangsu 226000 China; 3 Department of Radiation Oncology, Affiliated Hospital of Nantong University, Nantong, 226000 China; 4 Department of General Surgery, Huaian Hospital of Huaian City, Huaian, Jiangsu 223200 China

**Keywords:** Hepatocellular carcinoma, Double quinone enzymes, USP14

## Abstract

**Background:**

Hepatocellular carcinoma (HCC) is a common malignant tumor with strong invasiveness and poor prognosis. Previous studies have demonstrated the significant role of USP14 in various solid tumors. However, the role of *USP14* in the regulation of HCC development and progression remains unclear.

**Methods:**

We discovered through GEO and TCGA databases that USP14 may play an important role in liver cancer. Using bioinformatics analysis based on the Cancer Genome Atlas (TCGA) database, we screened and identified *USP14* as highly expressed in liver cancer. We detected the growth and metastasis of HCC cells promoted by *USP14* through clone formation, cell counting kit 8 assay, Transwell assay, and flow cytometry. In addition, we detected the impact of *USP14* on the downstream protein kinase B (AKT) and epithelial-mesenchymal transition (EMT) pathways using western blotting. The interaction mechanism between USP14 and HK2 was determined using immunofluorescence and coimmunoprecipitation (CO-IP) experiments.

**Results:**

We found that sh-USP14 significantly inhibits the proliferation, invasion, and invasion of liver cancer cells, promoting apoptosis. Further exploration revealed that sh-USP14 significantly inhibited the expression of *HK2*. Sh-USP14 can significantly inhibit the expression of AKT and EMT signals. Further verification through immunofluorescence and CO-IP experiments revealed that *USP14* co-expressed with *HK2*. Further research has found that *USP14* regulates the glycolytic function of liver cancer cells by the deubiquitination of HK2. USP14 regulates the autophagy function of liver cancer cells by regulating the interaction between SQSTM1/P62 and HK2.

**Conclusions:**

Our results indicate that *USP14* plays a crucial role in the carcinogenesis of liver cancer. We also revealed the protein connections between USP14, HK2, and P62 and elucidated the potential mechanisms driving cancer development. The USP14/HK2/P62 axis may be a new therapeutic biomarker for the diagnosis and treatment of HCC.

**Supplementary Information:**

The online version contains supplementary material available at 10.1186/s12885-024-12009-y.

## Background

Hepatocellular carcinoma (HCC), the predominant primary liver malignancy and the third most prevalent cause of cancer mortality, is currently managed through surgical resection and liver transplantation, which are considered the most efficacious treatment modalities [1]. Despite these interventions, the five-year survival rate of patients diagnosed with liver cancer remains considerably low [2–4]. While prior investigations have demonstrated the involvement of epigenetic alterations and molecular changes within established signaling pathways in the genesis and progression of HCC, further exploration of the relevant molecular mechanisms is still needed. Further research is warranted in this area [5]. Therefore, it is necessary to elucidate the potential molecular mechanisms of HCC and discover new potential targets to prevent its occurrence and development.

Ubiquitin specific protease 14 (USP14) is a member of the ubiquitin specific protease (USP) protein family [6, 7]. It interacts with 26 S proteasome complexes and enhances deubiquitination by reversibly binding to Rpn1 in proteasome 19 S regulatory granules [8, 9]. Multiple studies have demonstrated the significant involvement of USP14 in tumor advancement [10, 11]. Additionally, several studies have identified an overexpression and amplification of USP14 in cancer, which has been correlated with a reduced survival rate [12, 13]. Currently, there is a lack of research investigating the expression and regulatory mechanisms of USP14 in liver cancer, and its specific role and molecular mechanisms in the initiation and progression of liver cancer. Consequently, This study focused on investigating the biological function of USP14 on HCC by choosing it as the research object. The study also explored the newly discovered interaction between USP14 and HK2 protein and further investigated how USP14 regulates the occurrence and development of liver cancer through HK2. This study provides new ideas for the treatment and research of liver cancer.

## Methods

### Cell culture and transfection

HCC cell lines, MHCC97H and HCCLM3, were procured from American Type Culture Collection (ATCC). The cells were cultured in Dulbecco’s modified Eagle medium (DMEM). Plasmids were introduced into the cells using Lipofectamine 3000 reagent (Invitrogen, Carlsbad, CA, USA), and transfection efficiency was analyzed by western blotting. The ensuing experiments were executed 24 h post-transfection.

### CCK-8 assay

To evaluate cell proliferation, we utilized the cell counting kit 8 (CCK8) technique. Hundred microliters of a cell suspension was placed into each well of a 96-well plate. Then, we introduced 10 µL of the CCK8 solution into every well. After incubating the culture plate for 4 h, the absorbance was measured at an optical density (OD) of 450 nm using a microplate reader. Each set of experiments comprised three replicates, including auxiliary wells.

### Colony formation assay

In each experimental group, the cells were evenly distributed in 6-well plates at a density of 2,000 cells per well. After 14 d, the cells were fixed with a 4% solution of paraformaldehyde for 30 min. Next, 0.1% crystal violet staining was applied for 20 min. Following a thorough rinse, photographs were taken, and the resulting cell colonies were quantified.

### Flow cytometric analysis

After 48 h of transfection, the cells were collected and subjected to double staining using an apoptosis kit (Invitrogen) containing Annexin (Vibrio cholerae-derived Filamentous Hemagglutinin Protein Conjugated with Fluorescein Isothiocyanate) (V-FITC) and propidium iodide (PI). The proportion of stained cells was determined using a cell cycle kit (Invitrogen) based on PI. Subsequently, the cells underwent treatment at ambient temperature for a duration of 15 min, followed by the addition of 400 µl of phosphate buffered saline (PBS).

### Wound-healing assay

A scratch was made using a sterile suction tip with a volume of 10 µl. scratches at 0 and 72 h were photographed and recorded using an optical microscope to calculate the cell mobility.

### Transwell assay

The digestion process was performed on the cells, which were then suspended in serum-free medium. Approximately 10,000 cells were introduced into the upper chamber, while the lower chamber was supplied with 500 µl of DMEM medium. After incubation, cells were stained with 0.1% crystal violet for 20 min.

### Glucose consumption and lactate production tests

To evaluate the consumption of glucose and production of lactate, we employed a glucose assay (Solarbio, Beijing, China) and lactate assay kits (Solarbio, Beijing, China), respectively. Subsequently, the values obtained were normalized to the total protein concentration using a bicinchoninic acid (BCA) protein assay kit (Solarbio, Beijing, China).

### Western blot analysis

After extraction, the protein sample was applied to the sample well and separated using constant-current electrophoresis. After transferring it to a membrane, the membrane was sealed with skim milk powder for 2 h. The following day, after rinsing with Tris-Buffered Saline with Tween 20 (TBST), the secondary antibody was added. Finally, an automatic chemiluminescence imaging system was used to capture the images.

### Cellular immunofluorescence

Paraformaldehyde was used to fix hepatoma cells, followed by permeation with 1% Trion for a duration of 15 min. Subsequently, sealing solution was added to seal the cells. The primary antibody was introduced and incubated overnight. The cells underwent a 2-h incubation with secondary antibody. Following the washing step, microscopic examination was conducted with the addition of 4’,6-diamidino-2-phenylindole (DAPI).

### Coimmunoprecipitation (CO-IP)

The total cell lysate was combined with 5 µg of primary antibody or IgG antibody overnight. Then, 5 µl of agarose A + G agarose (Absin, Shanghai, China) was added and further incubated for 2 h at 4 °C. The protein-antibody complex was rinsed three times with PBS. Proteins were detected by sodium dodecyl sulfate-polyacrylamide gel electrophoresis and western blotting.

### Statistical analysis

Statistical analysis was performed using SPSS 26.0 software, and the data were computed as the average ± variation (± s). A comparison between the two data groups was conducted using an independent sample t test, and statistical significance was set at a significance level of *P* < 0.05.

## Results

### Predicting high expression of USP14 in liver cancer through bioinformatics analysis

**Fig. 1:**
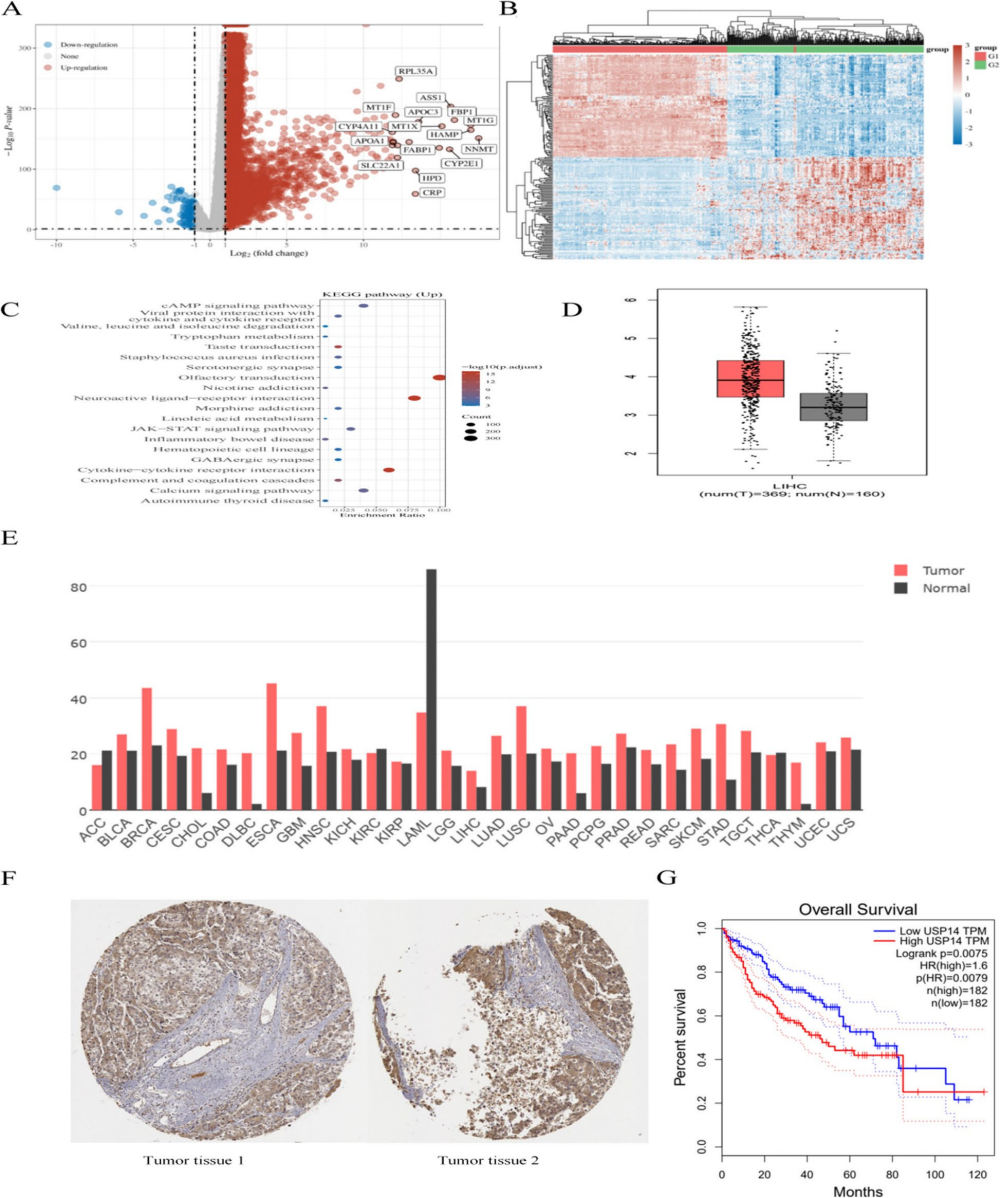
Predicting high expression of USP14 in liver cancer through bioinformatics analysis (**A**) Volcano map of differential expression between tumor tissue and control group in the GSE2509 core. **B** Heat map of differential expression between tumor tissue and control group in the GSE2509 core. **C** Kyoto Encyclopedia of Genes and Genomes (KEGG) analysis. **D** Analysis of the expression of *USP14* in liver cancer through Gene Expression Profiling Interactive Analysis (GEPIA). **E** Analyze the expression of *USP14* in different tumors through the Cancer Genome Atlas (TCGA) database. **F** Analyze the expression of *USP14* in liver cancer through the Proteinatlas database. **G** Prognosis of molecular *USP14* in liver cancer through TCGA database

First, we conducted a differential expression analysis using the GSE25097 dataset from the Gene Expression Omnibus (GEO) database. A volcano plot (Fig. 1A) and heat maps (Fig. 1B) revealed that 8,062 genes were upregulated and 114 genes were downregulated. Additionally, we identified differentially expressed mRNAs (DEmRNAs) in patients with HCC. Kyoto Encyclopedia of Genes and Genomes (KEGG) analysis indicated that these DEmRNAs were involved in the cAMP signaling pathway (Fig. 1C). Furthermore, we confirmed through the Gene Expression Profiling Interactive Analysis (GEPIA) database that USP14 is highly expressed in liver cancer (Fig. 1D). Differential expression analysis was also performed using TCGA database, which showed significant overexpression of USP14 in tumors such as breast cancer(BRCA), and liver cancer(LIHC) compared to normal tissues (Fig. 1E). Additionally, the Proteinatlas database (Fig. 1F) (https://www.proteinatlas.org/) revealed significant expression of USP14 in cancerous tissues. Our investigation of TCGA database highlighted a strong correlation between USP14 and the prognosis of individuals diagnosed with liver cancer, where increased levels of USP14 were consistently linked to a worsened prognosis (Fig. 1G).

### Expression and localization of USP14 in liver cancer cell lines

**Fig. 2:**
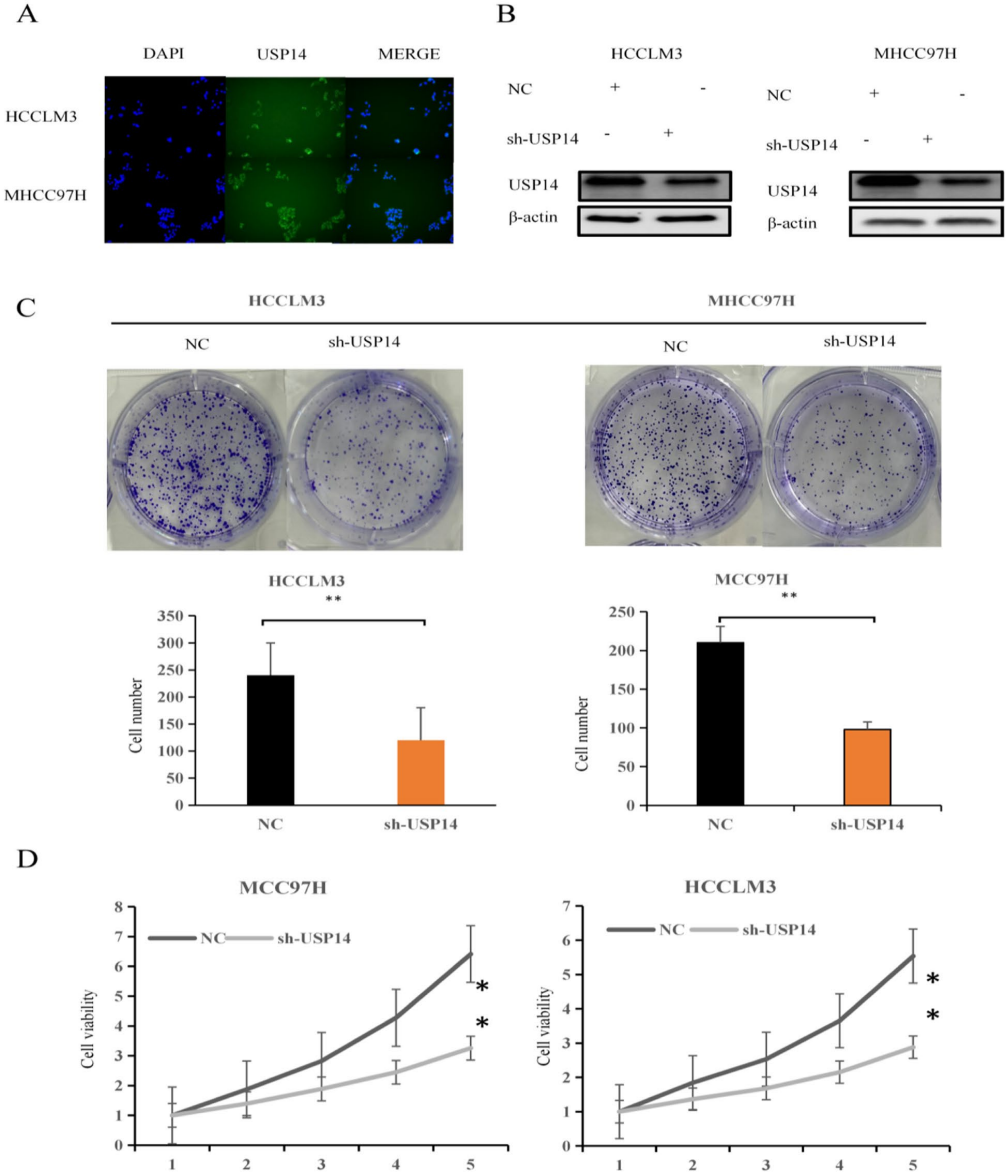
Expression of USP14 in liver cancer cells and its effect on proliferation (**A**) Localization of *USP14* expression in liver cancer cells through cellular immunofluorescence assay. **B **Western blot was used to detect the transfection effect of *USP14* in liver cancer cell lines. **C** Clone formation experiment and (**D**) cell counting kit 8 (CCK-8) assay were used to detect the effect of sh-USP14 on growth

To investigate the expression of USP14 in liver cancer tissues, immunofluorescence was used to detect its expression in the MHCC97H and HCCLM3 cells. The results revealed that USP14 was expressed in both the nucleus and cytoplasm of liver cancer cells (Fig. 2A).

### Western blot was used to detect the transfection effect of USP14 in liver cancer cell lines

To assess the effect of USP14 on the biological behavior of liver cancer cell lines, Lipofectamine 3000 was used for transient transfection. sh-USP14 was transfected into MHCC97H and HCCLM3 liver cancer cell lines, and western blotting was performed to measure the expression of USP14. The findings demonstrated that compared to the empty group, MHCC97H and HCCLM3 cells transfected with sh-USP14 exhibited significant downregulation of sh-USP14 expression (Fig. 2B).

### Effect of USP14 on the growth of liver cancer cells

Using clone formation experiment and CCK-8 assays, we determined the effect of sh-USP14 on cell growth. The clone formation assay showed that the size of the colonies in the sh-USP14 cells was smaller than that in the NC group (Fig. 2C). Similarly, the CCK-8 assay revealed that sh-USP14 significantly suppressed the proliferation of MHCC97H and HCCLM3 cells compared to that in the NC group (Fig. 2D). The results obtained from CCK-8 and clone formation experiments provide substantial evidence indicating that the overexpression of USP14 hampers the growth of liver cancer cells.

### Effect of USP14 on cell cycle and apoptosis of liver cancer cells

**Fig. 3:**
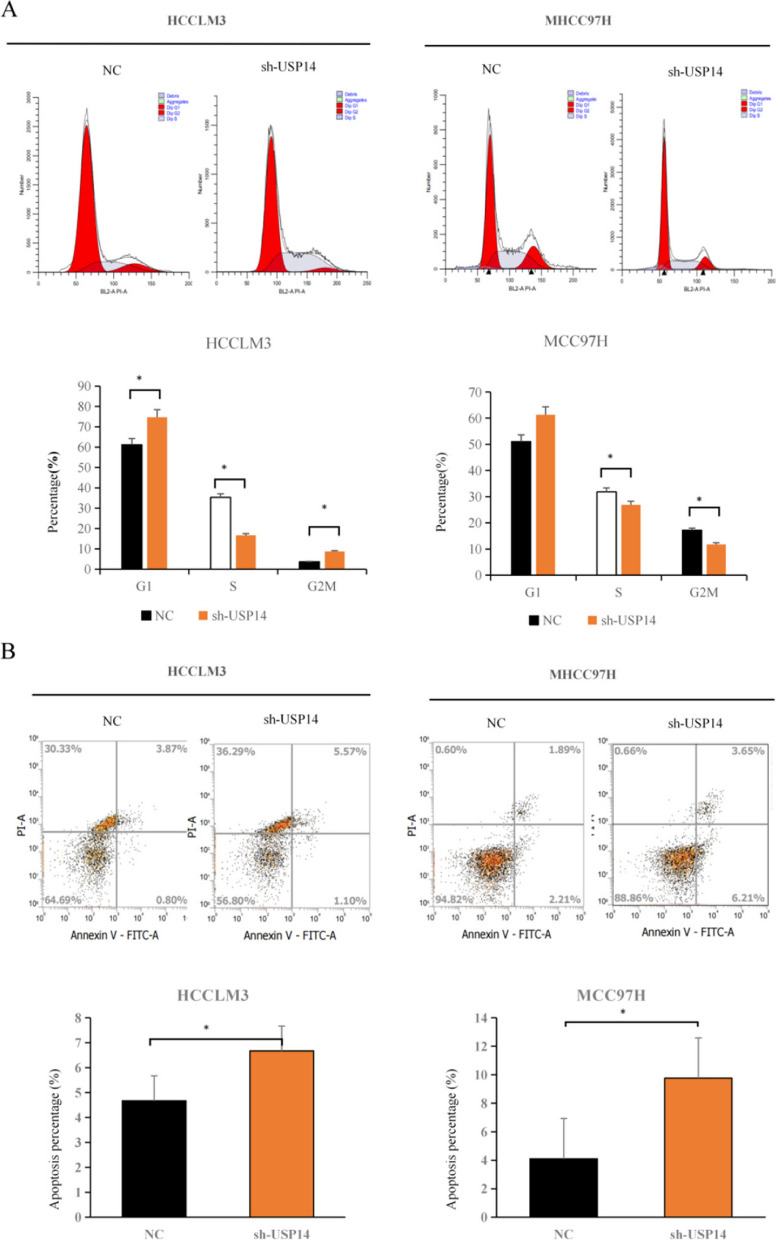
Effect of USP14 on cell cycle and apoptosis of liver cancer cells (**A**) Analyzing the distribution of the *USP14* gene cycle in MHCC97H and HCCLM3 cells through flow cytometry analysis. **B **Analyzing the effect of the *USP14* gene cycle distribution on apoptosis in MHCC97H and HCCLM3 cells through flow cytometry analysis

To examine the effect of sh-USP14 on cell cycle progression and programmed cell death, flow cytometry was performed. The observations revealed a substantial augmentation in the G1 phase cellular population in the sh-USP14 group compared with that in the NC group. Moreover, a notable reduction in S-phase cell population was observed in the sh-USP14 group. These findings demonstrated a robust association between *USP14* and the distribution of MHCC97H and HCCLM3 cells across the cell cycle (Fig. 3A). Additionally, by reducing the expression of *USP14* in MHCC97H and HCCLM3 cells, a marked increase in the number of cells undergoing programmed cell death was observed, as assessed by flow cytometry (Fig. 3B). In summary, these findings suggested that sh-USP14 effectively enhanced programmed cell death in liver cancer cells.

### Effects of USP14 on migration and invasion of hepatocellular carcinoma

**Fig. 4:**
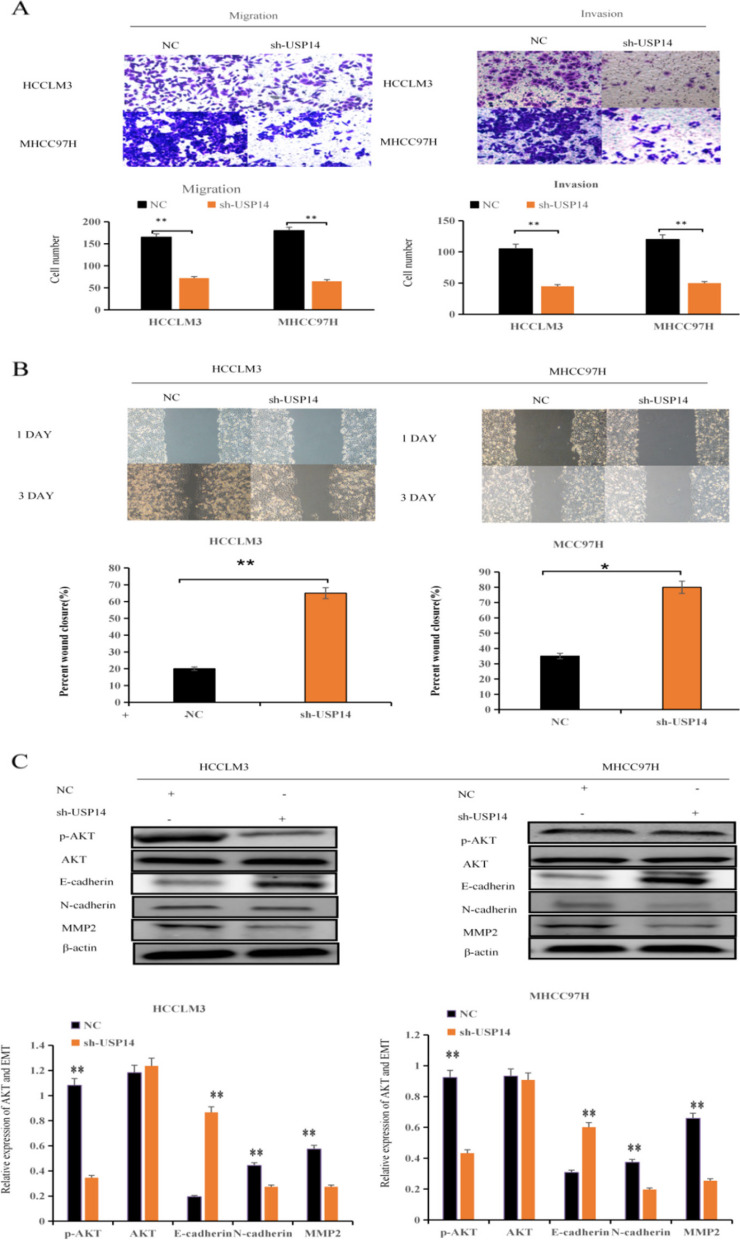
Effects of USP14 on migration and invasion of hepatocellular carcinoma cell migration and signaling pathways (**A**) Effect of *USP14* on the migration and invasion of hepatocellular carcinoma cell migration was detected by the Transwell test. **B** Effect of *USP14* on the migration of hepatocellular carcinoma cell migration was detected by the scratch test. **C** Detection of p-AKT/AKT, E-cadherin, N-cadherin, and MMP2 expression through western blot

To analyze the influence of *USP14* on the movement and infiltration capabilities of HCC cells, we performed transwell experiments (Fig. 4A) and wound healing assessments (Fig. 4B). These results consistently revealed that the overexpression of *USP14* hindered the mobility and penetration of MHCC97H and HCCLM3 cells.

### Effect of USP14 on AKT and EMT signaling pathways

To investigate the effect of *USP14* on the proliferation, invasion, and migration of HCC cells, MHCC97H and HCCLM3 cells were transfected with sh-USP14 or sh-NC. Subsequently, the expression levels of p-AKT/AKT, E-cadherin, N-cadherin, and MMP2 were analyzed using western blotting. The results revealed substantial suppression of p-AKT, N-cadherin, and MMP2 expression upon sh-USP14 treatment, along with the enhancement of E-cadherin expression (Fig. 4C). These findings indicate that the overexpression of *USP14* has the potential to hinder liver cancer cell proliferation, invasion, and migration via the AKT and EMT signaling pathways.

### USP14 promotes glycolysis through HK2

**Fig. 5:**
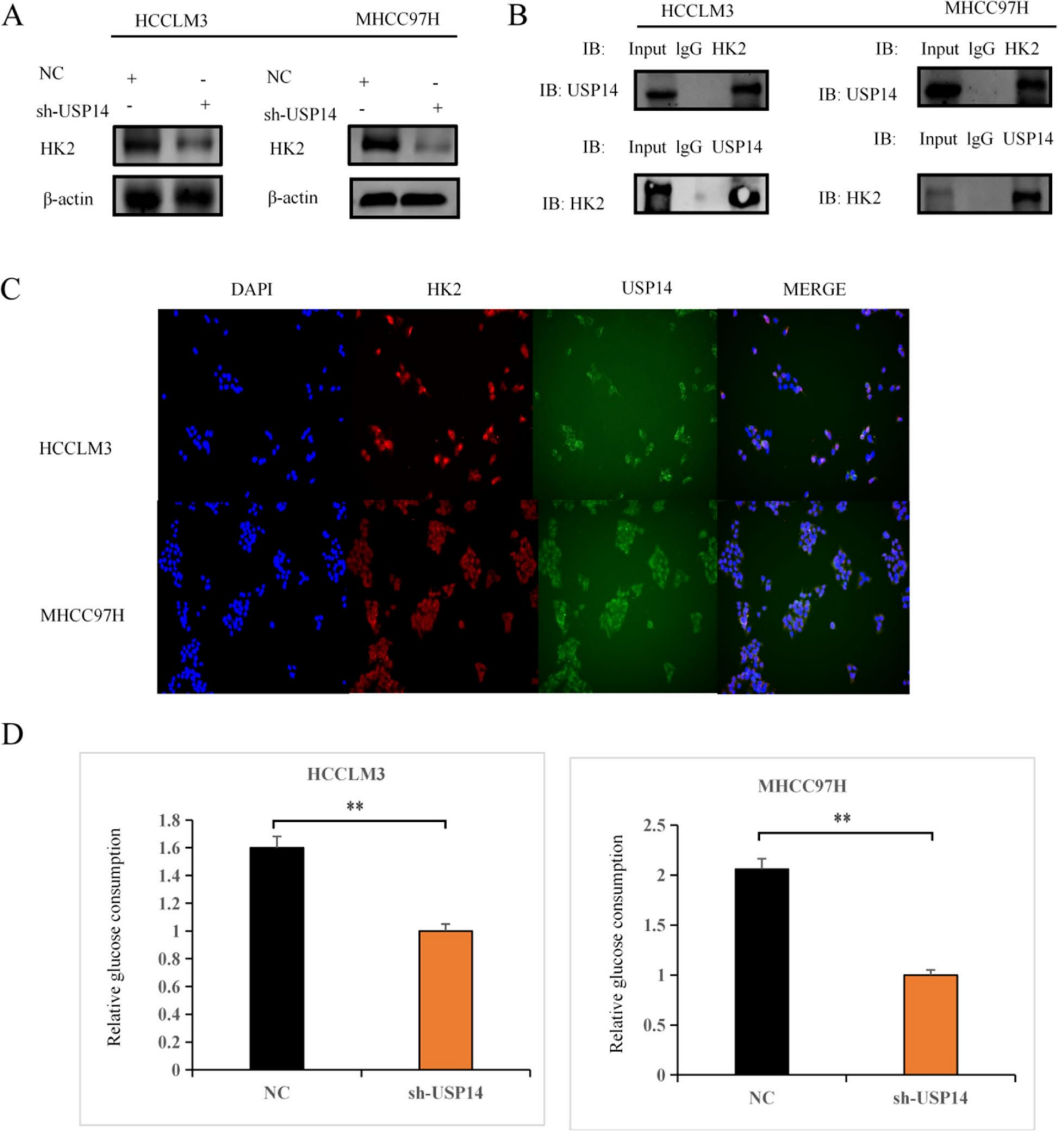
USP14 promotes glycolysis through HK2 (**A**) The effect of knocking down *USP14* on the protein expression of HK2. **B** Localization of HK2 expression in liver cancer cells through cellular immunofluorescence assay. **C** Detection of the interaction between USP14 and HK2 at the protein level through coimmunoprecipitation. **D** Glucose consumption and (**E**) lactate production were used to detect *USP14* knockdown in MHCC97H and HCCLM3 cells

In MHCC97H and HCCLM3 cells, the protein level of HK2 decreases after *USP14* knockdown (Fig. 5A). Fluorescence analysis revealed that HK2 was predominantly located in the cytoplasm of MHCC97H and HCCLM3 cells (Fig. 5B). CO-IP results demonstrated an interaction between USP14 and HK2 at the protein level (Fig. 5C). Experiments on glucose consumption (Fig. 5D) and lactate production (Fig. 5E) showed that *USP14* knockdown increased glucose uptake and lactate production in MHCC97H and HCCLM3 cells. These findings suggested that USP14-induced aerobic glycolysis was dependent on HK2.

### USP14 mediates the ubiquitination and stability of HK2

**Fig. 6:**
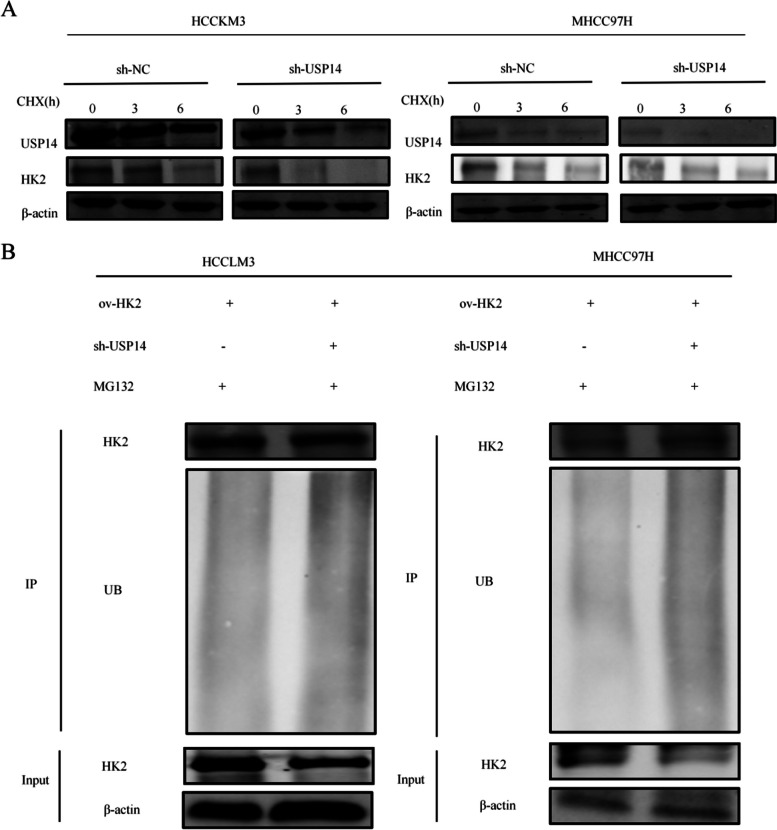
USP14 mediated deubiquitination and stability of HK2 (**A**) Observing the effect of sh-USP14 on the degradation of HK2 in MHCC97H and HCCLM3 cells after knockdown of *USP14* using CHX (a protein synthesis inhibitor). **B** Ubiquitination of HK2 after knocking down *USP14*

We treated USP14 knockdown MHCC97H and HCCLM3 cells with the protein synthesis inhibitor CHX. Our findings showed that sh-USP14 accelerated the degradation of HK2 in both the cell lines (Fig. 6A). Ubiquitination of HK2 was increased in *USP14* knockdown cells (Fig. 6B). These results suggested that *USP14* plays a role in mediating HK2 deubiquitination and stability.

#### USP14 relies on HK2 mediated autophagy

**Fig. 7:**
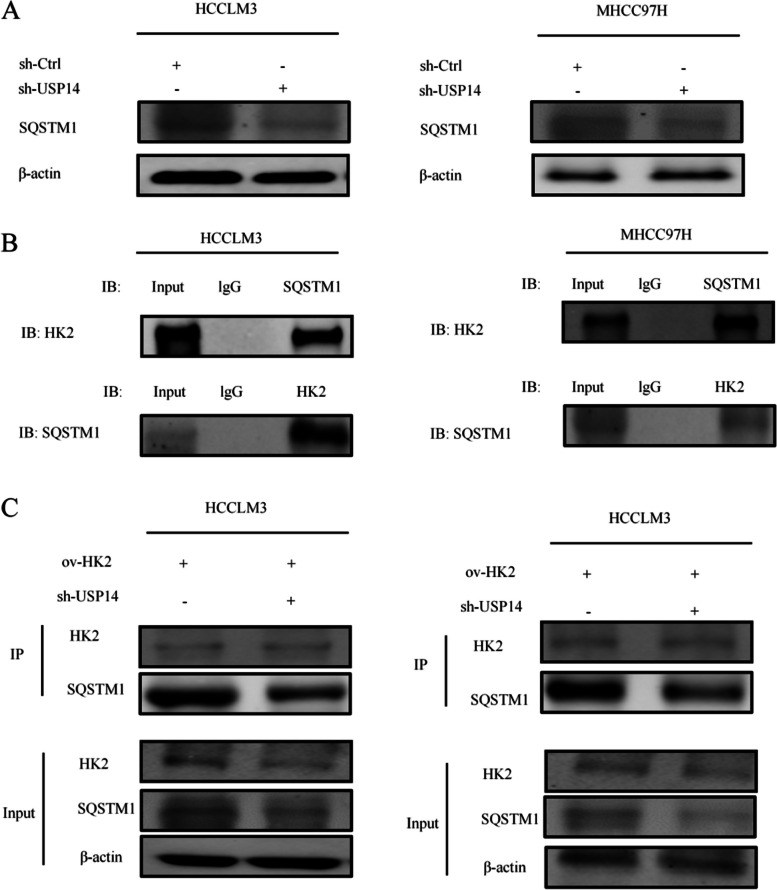
USP14 regulates autophagy by regulating the expression of SQSTM1/P62 through HK2 (**A**) In MHCC97H and HCCLM3 cells, the protein levels of SQSTM1/P62 decreased after knocking down *USP14*. **B** Detection of the interaction between SQSTM1/P62 and HK2 at the protein level through coimmunoprecipitation (CO-IP). **C** CO-IP experiments show that knocking down *USP14* weakens the interaction between SQSTM1/P62 and HK2

In addition, the protein levels of SQSTM1/P62 were decreased in MHCC97H and HCCLM3 cells after *USP14* knockdown (Fig. 7A). Simultaneous CO-IP experiments indicated an interaction between SQSTM1/P62 and HK2 at the protein level (Fig. 7B). Furthermore, CO-IP experiments revealed that *USP14* knockdown weakened the interaction between SQSTM1/P62 and HK2 cells (Fig. 7C). These findings suggested that *USP14* regulates autophagy by influencing the expression of SQSTM1/P62 through HK2.

### Effects of USP14 on the proliferation, invasion, migration, and AKT/SQSTM1 pathway of liver cancer cells through HK2

**Fig. 8:**
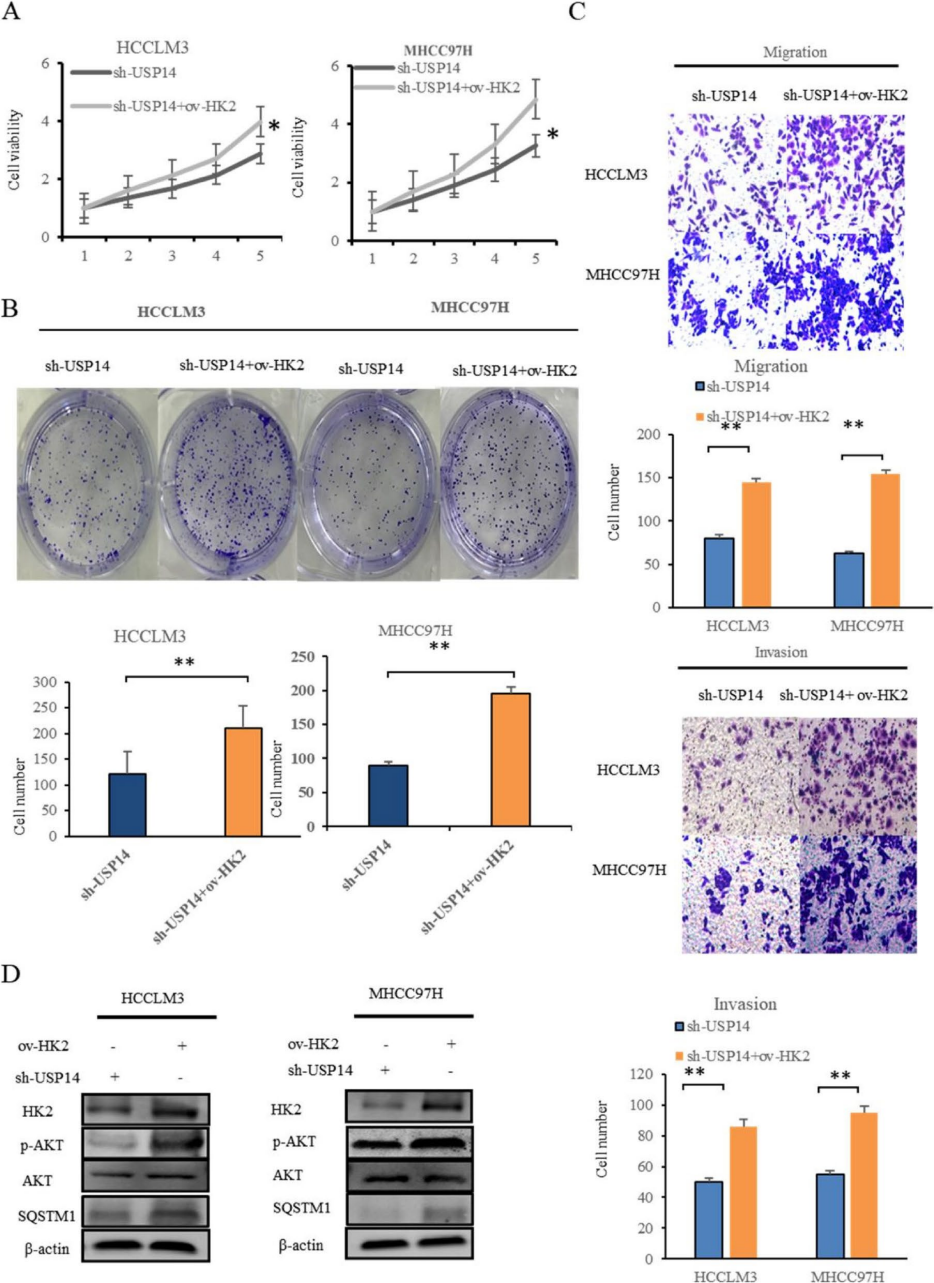
Effects of USP14 on the proliferation, invasion, migration, and AKT/SQSTM1 pathway of liver cancer cells (**A**) Overexpression of HK2 partially eliminated the inhibitory effects of sh-USP14 on cell proliferation through the cell counting kit 8 (CCK8) assay. **B** Overexpression of *HK2* partially eliminated the inhibitory effects of sh-USP14 on cell proliferation. **C** Overexpression of *HK2* partially eliminated the inhibitory effects of sh-USP14 on cell invasion and migration through Transwell experiments. **D** Overexpression of *HK2* can partially restore the inhibitory effect of *USP14* on p-AKT and SQSTM1

To investigate the role of *USP14* in promoting the proliferation, invasion, and migration of MHCC97H and HCCLM3 cells through HK2, we conducted a recovery experiment to restore the inhibited expression of HK2 by sh-USP14. Our findings from the CCK8 (Fig. 8A), clone formation (Fig. 8B), and Transwell assays (Fig. 8C) revealed that the overexpression of HK2 partially reversed the inhibitory effects of sh-USP14 on cell proliferation, invasion, and migration. These results suggested that sh-USP14 inhibited the proliferation, invasion, and migration of MHCC97H and HCCLM3 cells via HK2. Furthermore, we investigated whether sh-USP14 inhibits the AKT and SQSTM1 signaling pathways in liver cancer cells by regulating HK2. Our response experiments demonstrated that the overexpression of HK2 partially restored the inhibitory effect of *USP14* on p-AKT and SQSTM1 (Fig. 8D).

## Discussion

In recent years, molecular targeted therapy for liver cancer has become a prominent area of research. Therefore, it is important to identify effective therapeutic targets for liver cancer [14].

*USP14* is a ubiquitinase that has emerged as a crucial regulatory factor in various diseases, including tumors, neurodegenerative disorders, and metabolic conditions, owing to its role in enhancing the stability of substrate proteins [15–17]. Previous investigations demonstrate USP14’s involvement in the progression of breast cancer by influencing key processes such as proliferation, invasion, and apoptosis [18]. It has been observed that limiting the activity of USP14 expedites the ubiquitination of K48 and promotes the degradation of androgen receptor (AR) protein facilitated by proteasomes. Furthermore, both genetic and pharmacological inhibition of *USP14* significantly suppressed the growth of AR-reactive breast cancer cells by impeding the G0/G1-S phase transition and inducing apoptosis [19]. However, the biological function and regulatory mechanism of USP14 in HCC are still unclear.

The results demonstrated that the knockdown of *USP14* resulted in decreased vitality, proliferation, migration, and invasion of liver cancer cells, while enhancing apoptosis. The results showed that the overexpression of liver cancer cells reduced their vitality, proliferation ability, migration, and invasion ability, and increased their ability to regulate apoptosis. Furthermore, this study explored the mechanism of *USP14* inhibition of liver cancer proliferation and invasion by examining its effect on the AKT and EMT signaling pathways. *USP14* knockdown significantly inhibits AKT and EMT signaling. Thus, *USP14* influences the growth, proliferation, and invasion of liver cancer cells, ultimately affecting the occurrence and development of liver cancer. This discovery provides new therapeutic targets for the treatment of liver cancer.

However, the specific mechanism through which *USP14* functions in liver cancer cells remains unclear. We discovered that USP14 is involved in the deubiquitination and stabilization of HK2, thereby regulating the proliferation, apoptosis, invasion, migration, and glycolysis of HCC. Numerous studies have consistently reported that the abnormal overexpression of HK2 is closely associated with cancer development, metastasis, and resistance to chemotherapy and radiotherapy [20]. High expression of HK2 has been observed in various types of human cancers and is associated with unfavorable prognosis in patients with cancer [21–23]. It is important to note that forced overexpression of HK2 triggers cancer cell invasiveness [24]. Recent studies have shown that PVT1 modulates the expression of HK2 by competing with endogenous miR-143 in gallbladder cancer (GBC) cells, which could provide valuable insights into the potential therapeutic targets for GBC at the molecular level [25]. Furthermore, HK2 has demonstrated its significance in a broad spectrum of cancer types [26, 27]. In this study, we discovered that *USP14* regulates autophagy by controlling the expression of SQSTM1/P62 via HK2. Autophagy plays a crucial role in regulating cell survival and maintaining homeostasis [28]. Previous studies demonstrated that autophagy and the Nrf2 system serve as primary cellular defense mechanisms against oxidative stress. Research has shown that the physical interaction between the autophagy linker p62 and the Nrf2 inhibitor Keap1 enhances its stability and transcriptional activity [29, 30].

To investigate the role of *USP14* in regulating liver cancer, we conducted an experiment focusing on the interaction between USP14 and HK2. Our findings revealed that the overexpression of HK2 partially counteracted the inhibitory effects of USP14 on various biological functions of hepatocellular carcinoma cells, including proliferation, invasion, migration, glycolysis, and apoptosis promotion. Furthermore, we observed that the overexpression of HK2 partially attenuated the inhibitory effect of USP14 on the AKT and P62 signaling pathways in hepatoma cells. These results provide further evidence that *HK2* acts as a target gene of USP14 at the cellular level, and that USP14 regulates liver cancer by influencing the HK2/AKT/P62 axis. This study serves as an experimental and theoretical foundation for understanding the development and mechanisms of *USP14* in liver cancer as well as for the subsequent treatment of this disease.

## Conclusions

*USP14* plays a crucial role in the progression and onset of HCC — a form of liver cancer. Our extensive analysis revealed that the activities of hepatoma cells, including proliferation, invasion, migration, apoptosis, glycolysis, and autophagy, were significantly affected by USP14 through its interaction with HK2. Notably, this study uncovered a previously unknown connection between the USP14 and HK2 proteins, elucidating the underlying mechanism driving the development of liver cancer. Identifying USP14 as a potential biomarker for HCC is of immense importance as it lays the groundwork for a deeper understanding of HCC pathogenesis. Although this study has a certain degree of innovation, our research still lacks in-depth research. In addition, this study did not explore any correlation with clinical practice, and it does not have clinical translational significance.

### Supplementary Information

**Supplementary Material 1.**

## Data Availability

The raw date supporting the conclusion of this article will be made available by authors, without undue reservation.

## Abbreviations

HCC: Hepatocellular carcinoma
CO-IP: Coimmunoprecipitation
TCGA: The Cancer Genome Atlas
CCK8: Cell counting kit 8
AKT: Protein kinase B
EMT: Epithelial-mesenchymal transition
UPS: Ubiquitin-proteasome system
DUBs: Double quinone enzymes
ATCC: American Type Culture Collection
DMEM: Dulbecco’s modified Eagle medium
V-FITC: Vibrio cholerae-derived Filamentous Hemagglutinin Protein Conjugated with Fluorescein Isothiocyanate
PI: Propidium iodide
PBS: Phosphate buffered saline
BCA: Bicinchoninic acid
TBST: Tris-Buffered Saline with Tween 20
DAPI: 4',6-diamidino-2-phenylindole
GEO: Gene Expression Omnibus
DEmRNAs: Differentially expressed mRNAs
KEGG: Kyoto Encyclopedia of Genes and Genomes
GEPIA: Gene Expression Profiling Interactive Analysis
GBC: Gallbladder cancer
