# Supplementary material for: USP14 exhibits high expression levels in hepatocellular carcinoma and plays a crucial role in promoting the growth of liver cancer cells through the HK2/AKT/P62 axis
Source: BMC Cancer. 2024 Feb 21;24:237. doi: 10.1186/s12885-024-12009-y (PMC10880281; doi:10.1186/s12885-024-12009-y)
Supplement: Supplementary file 1 — Supplementary Material 1. [file 12885_2024_12009_MOESM1_ESM.pptx]

## Slide 1
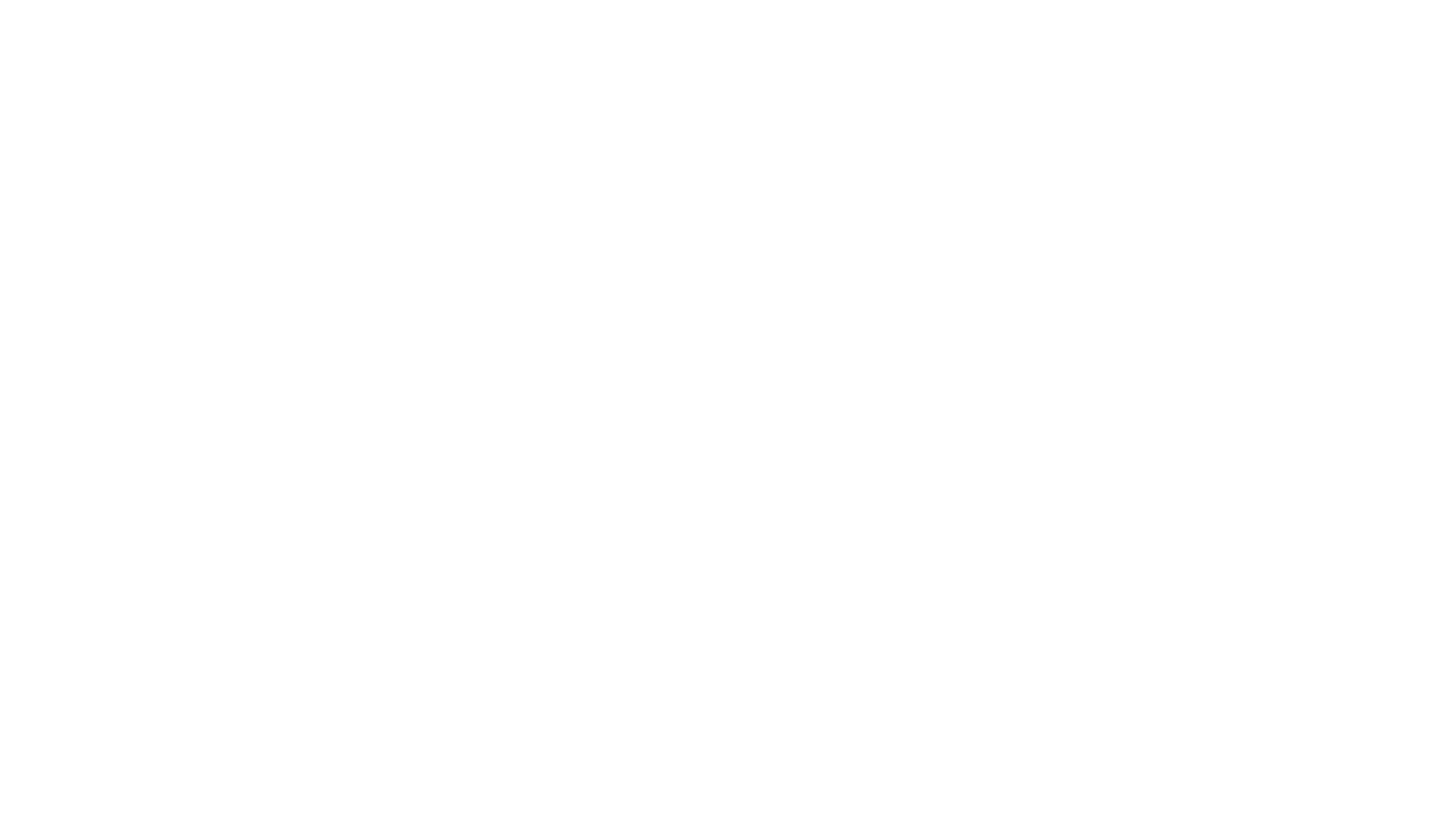

#

## Slide 2
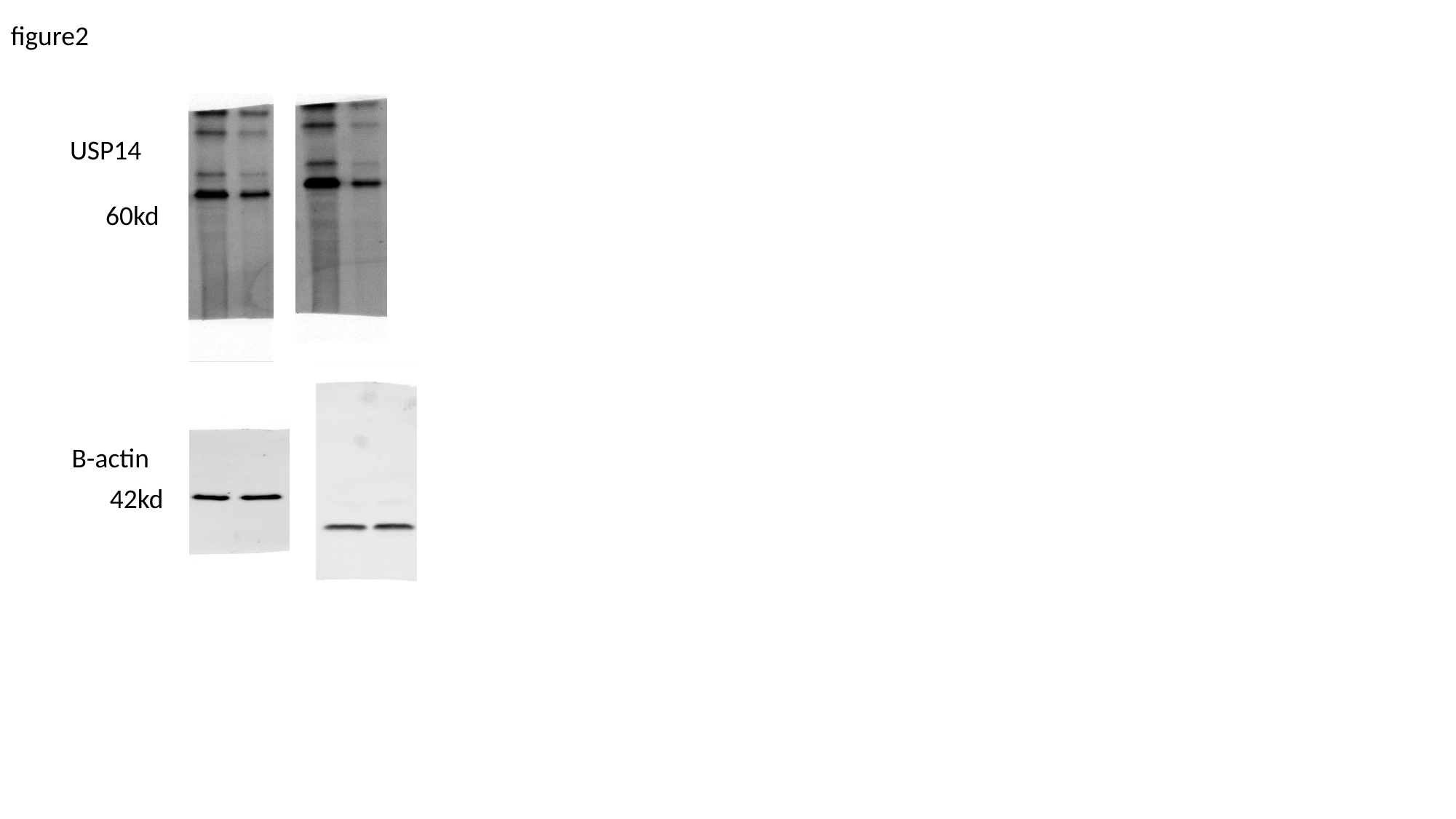

figure2
USP14
60kd
Β-actin
42kd

## Slide 3
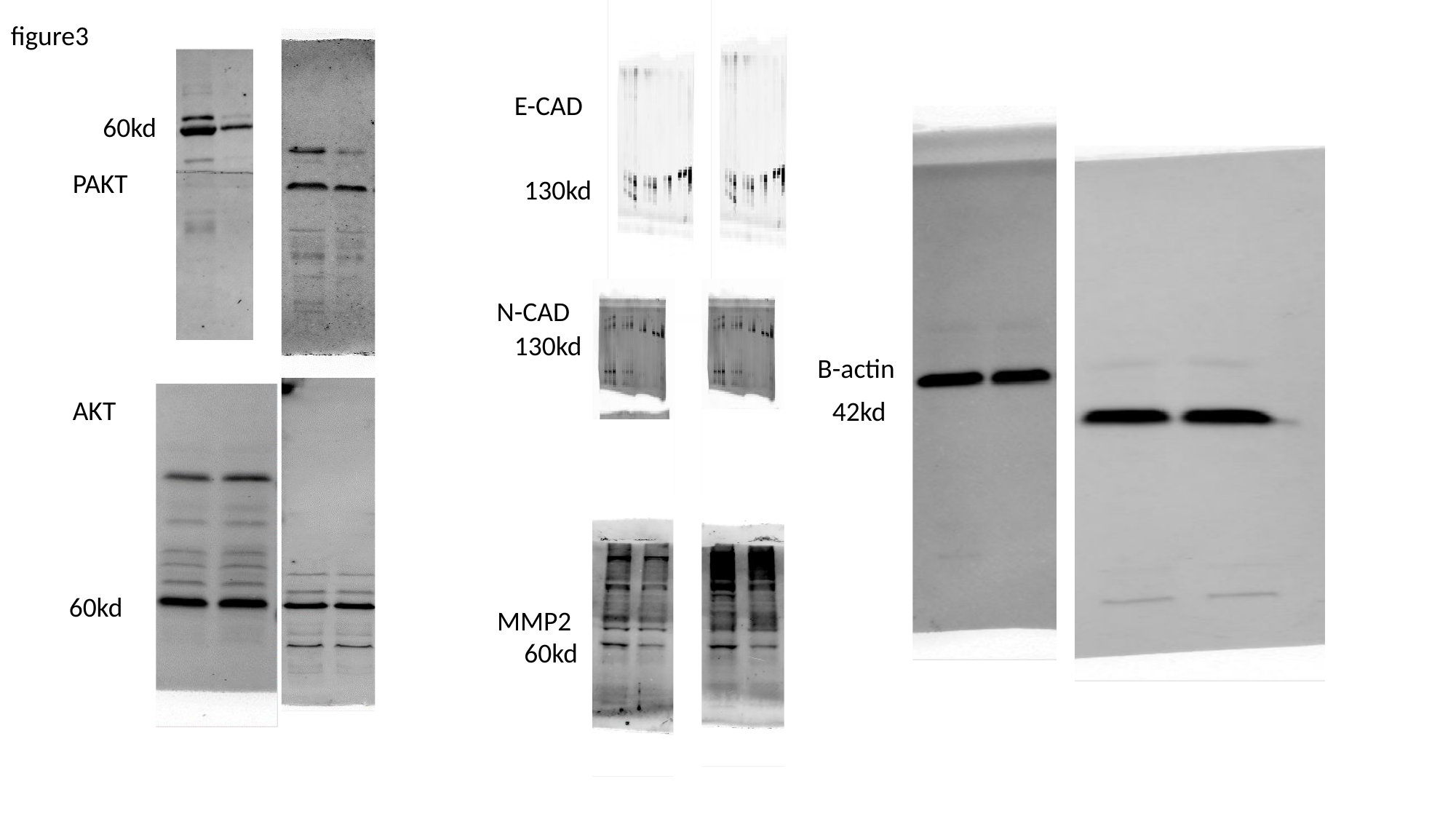

figure3
E-CAD
60kd
PAKT
130kd
N-CAD
130kd
Β-actin
AKT
42kd
60kd
MMP2
60kd

## Slide 4
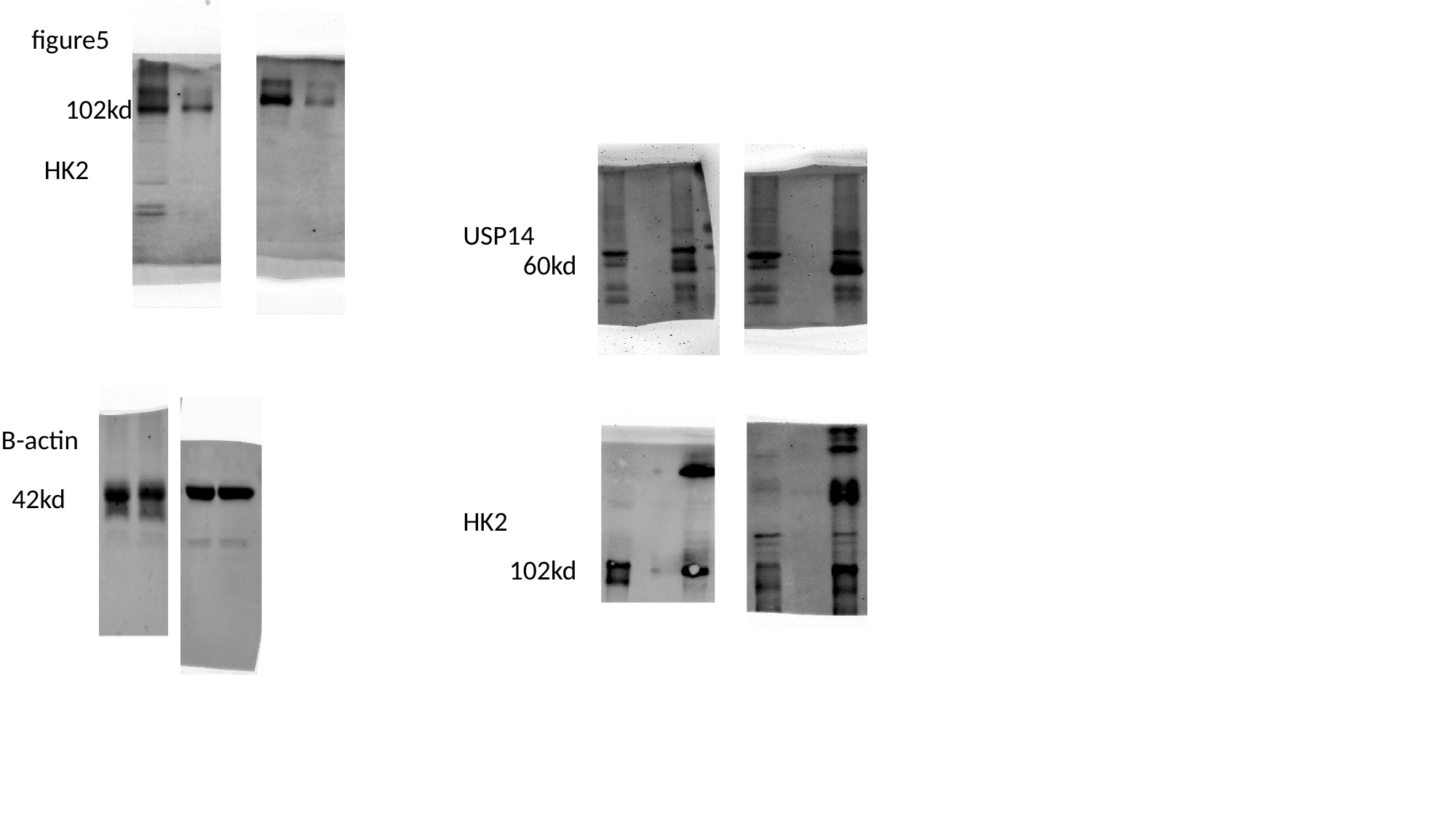

figure5
102kd
HK2
USP14
60kd
Β-actin
42kd
HK2
102kd

## Slide 5
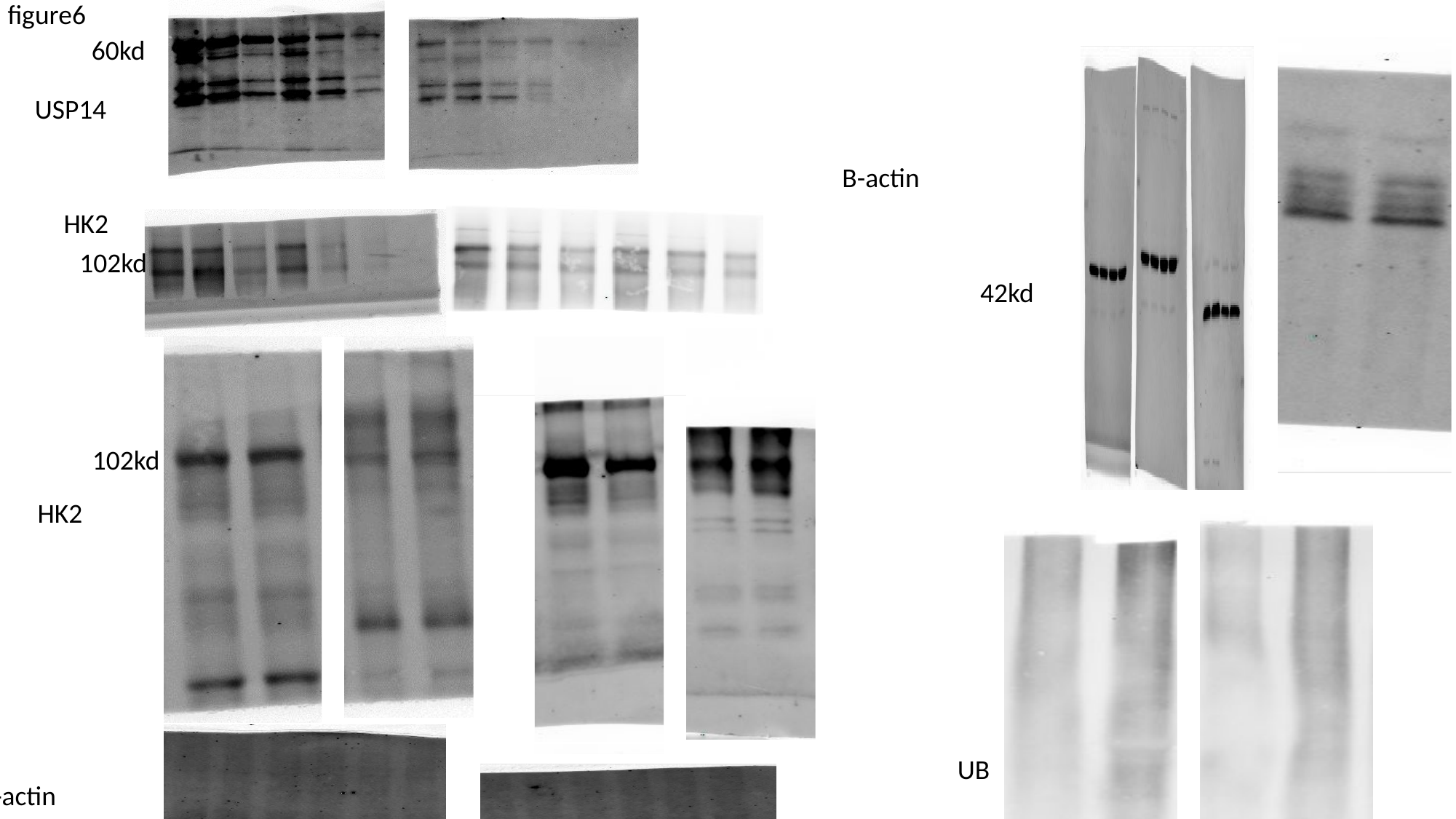

figure6
60kd
USP14
Β-actin
HK2
102kd
42kd
102kd
HK2
UB
Β-actin
42kd

## Slide 6
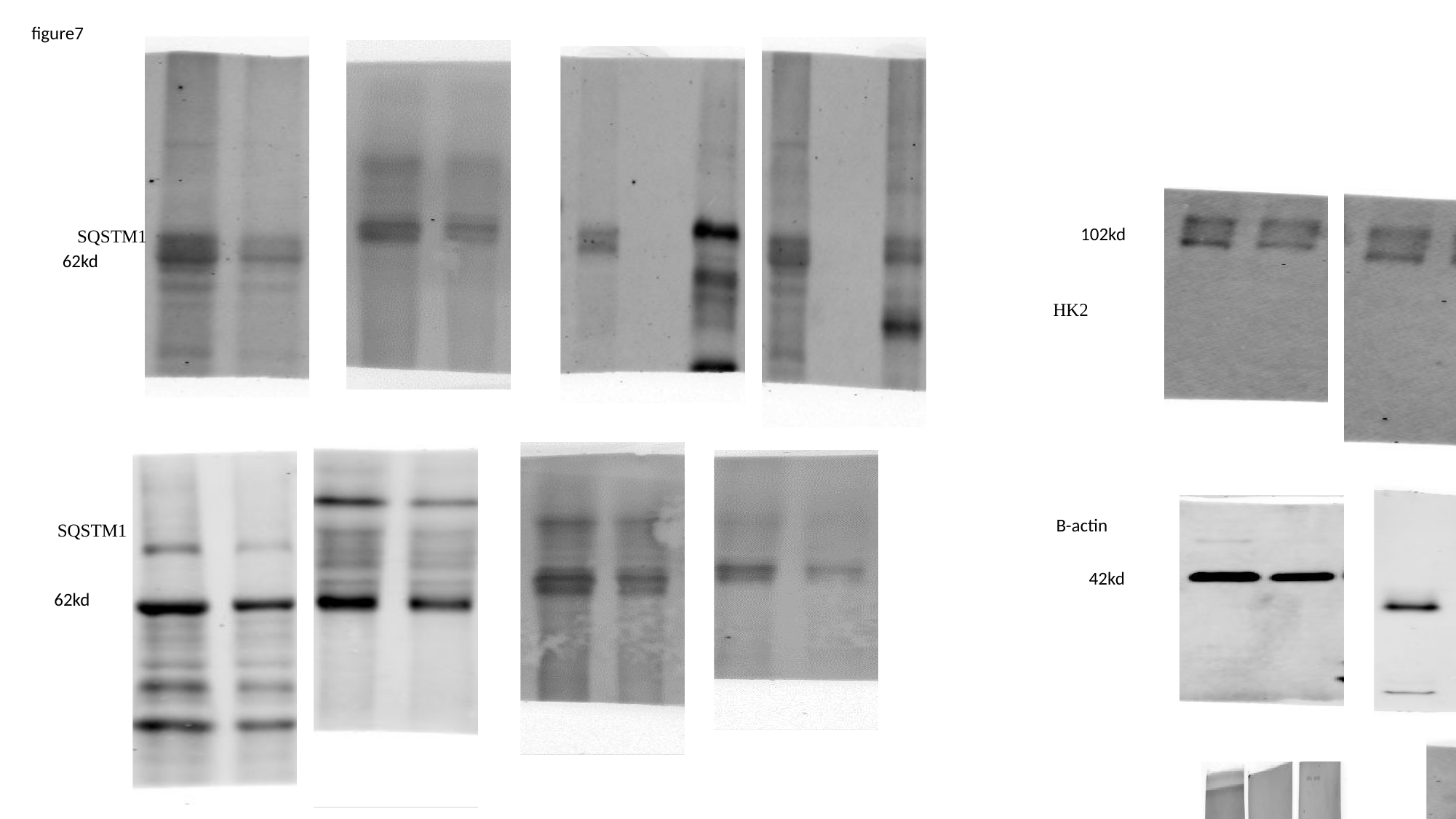

figure7
102kd
SQSTM1
62kd
HK2
Β-actin
SQSTM1
42kd
62kd
42kd
figure7
HK2
102kd

## Slide 7
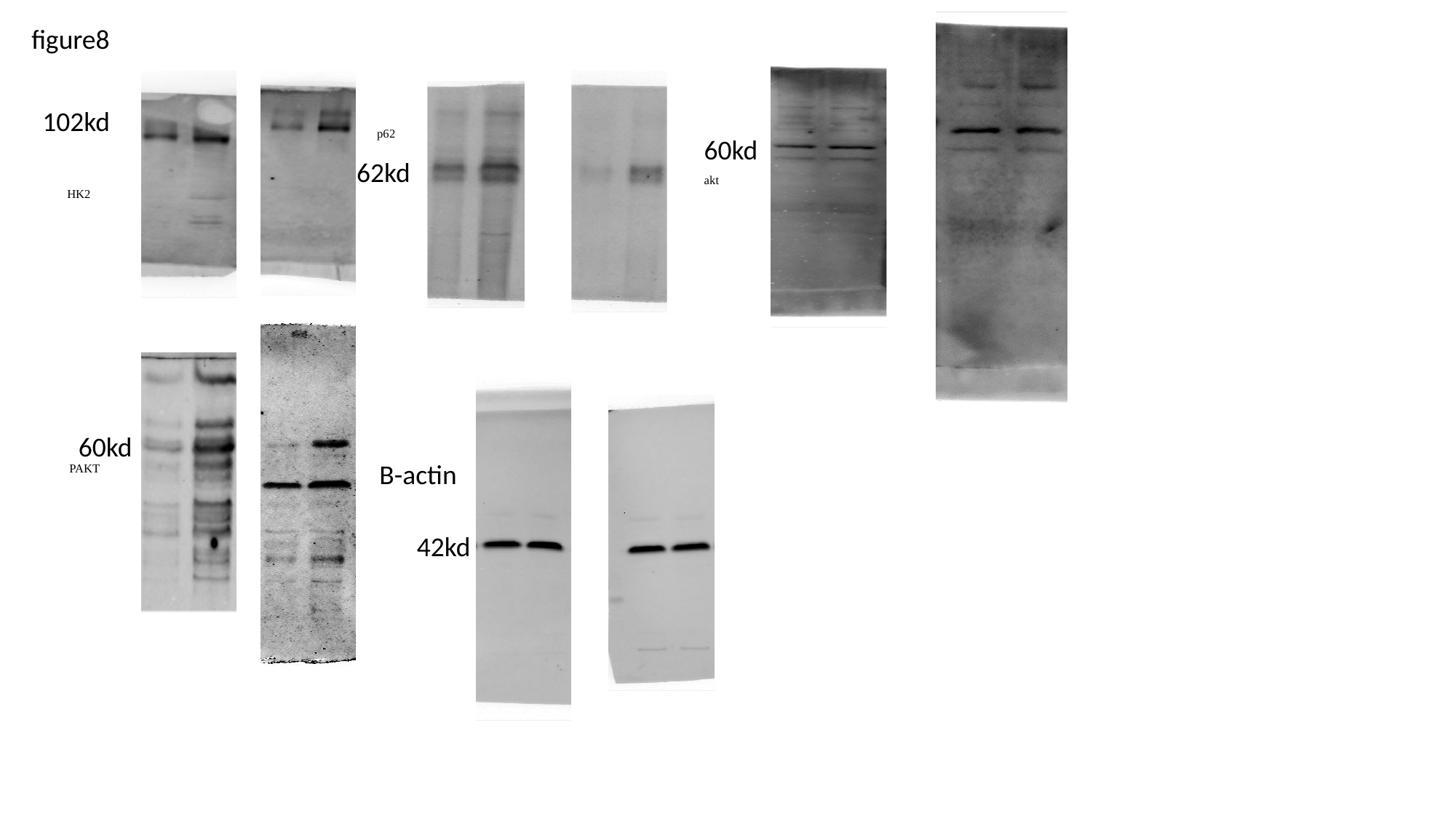

figure8
102kd
p62
60kd
62kd
akt
HK2
60kd
Β-actin
PAKT
42kd
